# Whole Genome Analysis of Selected Human Group A Rotavirus Strains Revealed Evolution of DS-1-Like Single- and Double-Gene Reassortant Rotavirus Strains in Pakistan During 2015–2016

**DOI:** 10.3389/fmicb.2019.02641

**Published:** 2019-11-14

**Authors:** Asma Sadiq, Nazish Bostan, Habib Bokhari, Kwe Claude Yinda, Jelle Matthijnssens

**Affiliations:** ^1^Department of Biosciences, COMSATS University (CUI), Islamabad, Pakistan; ^2^Department of Microbiology and Immunology, Rega Institute for Medical Research, Laboratory of Viral Metagenomics, KU Leuven, Leuven, Belgium

**Keywords:** morbidity, mortality, reassortant, genotype constellation, surveillance

## Abstract

Acute gastroenteritis due to group A rotaviruses (RVAs) is the leading cause of infant and childhood morbidity and mortality particularly in developing countries including Pakistan. In this study we have characterized the whole genomes of five RVA strains (PAK56, PAK419, PAK585, PAK622, and PAK663) using the Illumina HiSeq platform. The strains PAK56 and PAK622 exhibited a typical Wa-like genotype constellation (G9-P[8]-I1-R1-C1-M1-A1-N1-T1-E1-H1 and G3-P[8]-I1-R1-C1-M1-A1-N1-T1-E1-H1, respectively), whereas PAK419, PAK585, and PAK663 exhibited distinct DS-1-like genotype constellations (G3P[4]-I2-R2-C2-M2-A2-N2-T1-E2-H2, G1P[8]-I2-R2-C2-M2-A2-N2-T2-E2-H2, and G3P[4]-I2-R2-C2-M2-A2-N2-T2-E2-H2, respectively). Despite their DS-1-like genotype constellation, strain PAK585 possessed the typical Wa-like G1P[8] genotypes, whereas both PAK419 and PAK663 possessed the G3 genotype. In addition, PAK419 also possessed the Wa-like NSP3 genotype T1, suggesting that multiple reassortments have occurred. On Phylogenetic analysis, all of the gene segments of the five strains examined in this study were genetically related to globally circulating human G1, G2, G3, G6, G8, G9, and G12 strains. Interestingly, the NSP2 gene of strain PAK419 showed closest relationship with Indian bovine strain (India/HR/B91), suggesting the occurrence of reassortment between human and bovine RVA strains. Furthermore, strains PAK419, PAK585, and PAK663 were closely related to one another in most of their gene segments, indicating that these strains might have been derived from a common ancestor. To our knowledge this is the first whole genome-based molecular characterization of human rotavirus strains in Pakistan. The results of our study will enhance our existing knowledge on the diversity and evolutionary dynamics of novel RVA strains including DS-1-like intergenogroup reassortant strains spreading in Asian countries including Pakistan, in the pre-vaccine era. Therefore, continuous surveillance is recommended to monitor the evolution, spread and genetic stability of novel reassortant rotavirus strains derived from such events.

## Introduction

Group A rotaviruses (RVAs) belong to the family *Reoviridae* and are responsible for severe dehydrating diarrhea in children less than 5 years of age and in many animal species worldwide (Tacharoenmuang et al., 2016). In spite of the universal implementation of two rotavirus vaccines (Rotarix and RotaTeq), in 2016, rotavirus was estimated to cause 128,500 deaths of children less than 5 years of age, of which 13,396 deaths occurred in South Asia (Troeger et al., 2018).

These non-enveloped viruses are triple layered with icosahedral symmetry and carry 11 segments of double stranded RNA encoding six structural proteins (VP1-4, VP6, and VP7) and five to six non-structural proteins (NSP1-NSP6) (Desselberger, 2014). A classification system for RVA was proposed for each of these 11 gene segments represented by the acronym Gx-P[x]-Ix-Rx-Cx-Mx-Ax-Nx-Tx-Ex-Hx where x is an integer, which represents the genotypes of the VP7-VP4-VP6-VP1-VP2-VP3-NSP1-NSP2-NSP3-NSP4-NSP5 gene segments, respectively (Matthijnssens et al., 2008, 2011a). The whole genome-based analysis is an excellent approach to figure out the evolutionary pattern of RVA strains (Matthijnssens et al., 2011b; Yinda et al., 2016). To date at least 36 G, 51 P, 26 I, 22 R, 20 C, 20 M, 31 A, 22 N, 22 T, 27 E, and 22 H genotypes have been assigned by the Rotavirus Classification Working Group (RCWG, 2018).

Two major RVA genotype constellations are dominant in humans. The human Wa- like genotype constellation is characterized as I1-R1-C1-M1-A1-N1-T1-E1-H1, and is usually found in combination with the genotypes G1P[8], G3P[8], G4P[8], G9P[8], G12P[6], or G12P[8], whereas the human DS-1-like genotype constellation is characterized as I2-R2-C2-M2-A2-N2-T2-E2-H2 and tends to be combined with G2P[4], G8P[4] or G8P[6] (Matthijnssens et al., 2008; Matthijnssens and Van Ranst, 2012; Ide et al., 2015; Komoto et al., 2016). The human Wa-like genotype share many genotypes with porcine RVA, whereas several of the DS-1 like genotypes have a common ancestors with bovine RVA strain (Matthijnssens et al., 2008; Sadiq et al., 2018). Another minor human genotype constellation (AU-1-like) has been detected in humans with the genotype constellation G3-P[9]-R3-C3-M3-A3-N3-T3-E3-H3. The human AU-1 like RVA genotype constellation is believed to be derived from feline and/or canine RVAs (Matthijnssens and Van Ranst, 2012).

The segmented nature and error-prone-RNA-dependent RNA polymerase, lacking proofreading activity, provide rotavirus with several evolutionary mechanisms including point mutation, recombination, reassortment and interspecies transmission (Komoto et al., 2016; Tacharoenmuang et al., 2016; Jere et al., 2018). The emergence of novel RVA genotypes in humans is probably the results of both interspecies transmission and reassortment (between/within human and animal strains) (Matthijnssens et al., 2009; Kirkwood, 2010; Komoto et al., 2014; Ide et al., 2015; Bwogi et al., 2017). Although intergenogroup reassortants are constantly being generated, it is believed that RVAs strains having a pure Wa-like or pure DS-1-like genotype constellation have an evolutionary fitness advantage (Heiman et al., 2008). However, the emergence of uncommon human intergenogroup reassortant such as DS-1-like G1P[8] strains, or other DS-1-like (backbone I2-R2-C2-M2-A2-N2-T2-E2-H2) strains in combination with genotypes G1/2/3/8 and/or P[8] were recently reported in Japan followed by reports from Thailand, Vietnam, Malawi, Philippine, Australia, Hungary, and Spain (Yamamoto et al., 2014, 2017; Arana et al., 2016; Komoto et al., 2016; Roczo-Farkas et al., 2016; Jere et al., 2018).

The first unusual DS-1-like G1P[8] strains (HC12016, NT004, and OH3506) were detected in Japanese children suffering from severe gastroenteritis (Fujii et al., 2014; Kuzuya et al., 2014; Yamamoto et al., 2014) and after that such uncommon strains (PCB-180, SKT-109, and SSKT-41) were reported in Thailand in 2013 (Komoto et al., 2010). In 2013, other DS-1-like intergenogroup reassortant strains (LS-04 and SKT-281) with genotypes G2P[8] and G3P[8] were also detected in Thailand and strain D388 having genotype G3P[8], in Australia (Komoto et al., 2016; Roczo-Farkas et al., 2016). Moreover, DS-1-like intergenogroup reassortant strains having G8P[8] genotypes were discovered in Thailand in 2013–2014 and later similar strains were detected in Vietnam in 2014–2015 (Hoa-Tran et al., 2016; Guntapong et al., 2017). Likewise, in 2015 DS-1-like G3P[8] strains were also recognized in Hungary, Spain including strains ERN8263 and SS96217158 (Arana et al., 2016; Dóró et al., 2016).

Most of these novel DS-1-like strains were not completely sequenced. Whole genome analysis is a reliable method for obtaining conclusive data on the origin of RVA strain, and for tracing its evolutionary pattern. During the last decade, research on RVA in Pakistan mainly focused on the detection of current RVA genotypes based on VP7 and VP4. To the best of our knowledge, this is the first time genomes of Pakistani RVA strains have been fully sequenced and characterized. In this study, the Illumina HiSeq platform was used to obtain the complete nucleotide sequences of the whole genomes of five Pakistani RVA strains.

## Materials and Methods

### Ethics Statement

The study was approved by the Ethical Committee of PIMS, Benazir Bhutto Shaheed Hospital (BBH) and Internal Review Board (IRB) of COMSATS Institute of Information Technology, Islamabad. Written informed consent for molecular characterization of RVAs strains were obtained from the parents/guardians of the study participants.

### RVA Strains

RVA strains, PAK56, PAK419, PAK585, PAK622, and PAK663, showed maximum diversity upon phylogenetic analysis were selected for whole genome sequencing. These strains were detected in five fecal samples from hospitalized children aged less than 10 years with severe gastroenteritis admitted to the emergency pediatrics department of BBH (Benazir Bhutto Hospital) in Rawalpindi, and PIMS (Pakistan Institute of Medical Sciences) in Islamabad. These stool samples were collected during RVA surveillance program in Pakistan in 2015–2016 which involved a total of 639 samples (Sadiq et al., 2019). Of the total 639 samples RVA was found positive in 171 (26.8%) of the samples. Stool samples containing above selected strains were kept at −80°C until use.

### Sample Preparation for NGS

Each sample was enriched for viral particles by the NetoVIR (Novel Enrichment Technique Of VIRomes) protocol (Conceição-neto et al., 2015). Briefly, fecal suspensions (10%) were prepared by adding 50 mg of fecal sample in 500 μl of sterile PBS in a clean 1.5 ml centrifuge tube. Fecal suspensions were subjected to homogenization for 1 min at 3000 rpm with a MINILYS homogenizer (Bertin Technologies, Montigny-le-Bretonneux, France) and then centrifuged at 17000 × *g* for 3 min. The supernatant was filtered through a 0.8 μm (PES) filter (Merck Millipore, Burlington, MA, United States) at 17000 × *g* for 1 min. After filtration samples were treated for 2 h at 37°C with a cocktail of 7 μl of 20× homemade buffer (1 M Tris, 100 mM CaCl2 and 30 mM MgCl2, pH = 8), 2 μL of benzonase (Novagen, Madison, WI, United States) and 1 μL of micrococcal nuclease (New England Biolabs, MA, United States). Reaction was stopped by adding 7 μl of 10 nM EDTA. Nucleic acid extraction was conducted by using the QIAamp^®^ Viral RNA Mini kit from Qiagen (Qiagen, Hilden, Germany) according to manufacturer instructions without adding carrier RNA.

### cDNA Library Preparation and Illumina Sequencing

cDNA synthesis was carried out and random PCR amplification for 17 cycles were performed using a Whole Transcriptome Amplification (WTA) Kit (Sigma-Aldrich) according to the manufacturer’s instructions. The PCR products were purified with the MSB Spin PCRapace spin columns (Stratec, Berlin, Germany). The libraries for Illumina sequencing were prepared using the Nextera XT Library Preparation Kit (Illumina, San Diego, CA, United States) according to the manufacturer’s guide. Library purification was performed using a 1.8 ratio of Agencourt AMPure XP beads (Beckman Coulter, Inc., Nyon, Switzerland). The quality check of the purified cDNA library was performed on 2100 Bioanalyzer (Agilent Technologies). The libraries were quantified with the KAPA Library Quantification kit (Kapa Biosystems) followed by sequencing on a HiSeq^TM^ 2500 platform (Illumina) for 300 cycles (2 × 150 bp paired ends).

### Genomic Assembly

The obtained raw reads were trimmed for quality and adapters, and assembled into contigs *de novo* using Trimmomatic and SPAdes, respectively (Bankevich et al., 2012; Bolger et al., 2014). Scaffolds were then classified using DIAMOND in sensitive mode (Buchfink et al., 2015). Contigs assigned to RVA were used to map the trimmed reads using the Burrows-Wheeler Alignment tool (BWA) (Li and Durbin, 2009). Open reading frames (ORF) were identified with ORF Finder analysis tool^1^.

### Genotyping and Phylogenetic Analysis

Genotypes for all eleven gene segments of RVA were determined by the RotaC v2.0 automated genotyping tool for RVA according to the guidelines proposed by the Rotavirus Classification Working Group (RCWG) (Maes et al., 2009). Multiple nucleotide-based sequence alignments were conducted using ClustalW in MEGA version 6.06 (Tamura et al., 2013). Reference sequences were retrieved from GenBank and phylogenetic trees were constructed by Maximum Likelihood method with kimura-2-parameter model in MEGA 6.06 (Tamura et al., 2013). The statistical reliability was checked using 1000 bootstrap replicates. Nucleotide and amino acid distances were calculated using the *P* Distance Model. Phylogenetic analysis were performed using appropriate reference strains in addition to the RVA discovered in this study.

### Nucleotide Sequence Accession Number

The nucleotide sequences were submitted to the GenBank under the following accession numbers: **(VP7)** MH182443–MH182446 and MK370887, **(VP4)** MH182439–MH182442 and MK370888, **(VP6)** MH170022–MH170026, **(VP1)** MH166401–MH166404 and MK412569, **(VP2)** MH166405–MH166409, **(VP3)** MH170017–MH170021, **(NSP1)** MH182447–MH182451, **(NSP2)** MH182452–MH182456, **(NSP3)** MH182457–MH182461, **(NSP4)** MH182462–MH182466, and **(NSP5)** MH182467–MH182471.

## Results

### Genotyping Based on Whole-Genome Sequencing

In order to gain insight into the genetic variability among strains PAK56, PAK419, PAK585, PAK622, and PAK663 and their genetic relatedness with other RVA strains worldwide, the full-genome sequences of these five strains were determined using the NetoVIR protocol in combination with NGS Illumina HiSeq sequencing. Full or nearly full length nucleotide sequences reads were obtained for all the 11 gene segments of the five human rotavirus strains via Illumina HiSeq platform. The contigs length and number of reads mapped against the *de novo* assembled RVA genome contigs are given in the Table 1.

**TABLE 1:** The contigs length and number of reads mapped to each contig belongs to rotavirus.

		**VP7**	**VP4**	**VP6**	**VP1**	**VP2**	**VP3**	**NSP1**	**NSP2**	**NSP3**	**NSP4**	**NSP5**
PAK56	Contig length	1061	2359	1356	3302	2729	2591	1566	1059	1074	750	664
	# reads mapped to contig	792113	1328605	768901	2363458	1203604	2476905	1551211	204835	703273	227668	36579
PAK419	Contig length	1058	2359	1356	3302	2684	2592	1566	1059	1074	751	805
	# reads mapped to contig	305666	841067	338399	1263771	1078719	794388	164998	124797	377118	57925	51591
PAK585	Contig length	1062	2359	1356	3302	2566	2591	1566	1059	1074	751	805
	# reads mapped to contig	94755	653844	413656	1312186	1046341	1083868	187570	92835	195887	112604	67837
PAK622	Contig length	1062	2359	1356	3302	2735	2558	1566	1055	1005	694	612
	# reads mapped to contig	1140	1441	651	2002	929	967	1124	255	973	50	27
PAK663	Contig length	1062	2359	1356	3302	2684	2586	1563	1059	1066	751	813
	# reads mapped to contig	164487	758319	148623	895893	723230	717845	129346	40418	137151	35628	13692

**FIGURE 1:**
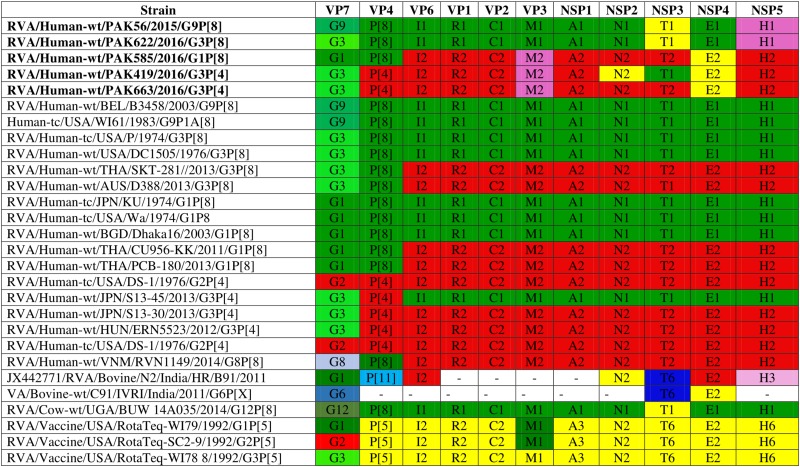
Comparison of the genotype constellation of 5 Pakistani strains (PAK56, PAK622, PAK585, PAK419 and PAK663) having the G9P[8], G3P[8], G1P[8], G3P[4], and G3P[4] genotypes, respectively, compared with those of selected human and animal strains. The green and red shading represent human Wa-like, and DS-1-like genotypes, respectively. Different shades of green are used for VP7 G genotypes mostly found in combination with the Wa-like genotype constellation. Yellow is used to indicate gene segments of bovine origin and purple gene segments of caprine origin.

Comparison of the complete genotype constellations of these strains with those of representative G1, G3, G9 and other RVA strains is shown in Figure 1. The eleven gene segments of two strains (PAK56 and PAK622) were found to have a typical Wa-like genotype constellation: G9-P[8]-I1-R1-C1-M1-A1-N1-T1-E1-H1 (PAK56) and G3-P[8]-I1-R1-C1-M1-A1-N1-T1-E1-H1 (PAK622). Three strains (PAK419, PAK585, and PAK663) comprised genotypes typical of both the Wa-like and DS-1-like genotype: G3-P[4]-I2-R2-C2-M2-A2-N2-T1-E2-H2, G1-P[8]-I2-R2-C2-M2-A2-N2-T2-E2-H2, and G3-P[4]-I2-R2-C2-M2-A2-N2-T2-E2-H2, respectively. The unusual genotype combination of strain PAK585 was similar to DS-1-like intergenogroup reassortant G1P[8] strains. On the other hand, strain PAK663 possessed a predominantly DS-1-like backbone (P[4]-I2-R2-C2-M2-A2-N2-T2-E2-H2) with the exception of the G3 genotype, most frequently encountered with a Wa-like genotype constellation. Strain PAK419 possessed a genotype constellation identical to PAK663, except for the T1 NSP3 genotype, also typical for the Wa-like genotype constellation.

The strains PAK56, PAK419, PAK585, PAK622, and PAK663 were named as RVA/Human-wt/PAK/PAK56/2015/G9P[8], RVA/Human-wt/PAK/PAK419/2016/G3P[4], RVA/Human-wt/PAK/PAK585/2016/G1P[8], RVA/Human-wt/PAK/PAK622/2016/G3P[8], and RVA/Human-wt/PAK/PAK663/2016/G3P[4], respectively following instructions for uniformity of RVAs naming by the RCWG.

### Phylogenetic Analysis

Phylogenetic trees were constructed for each of the 11 segments of the five RVA strains obtained in this study, together with representative strains from GenBank.

**FIGURE 2:**
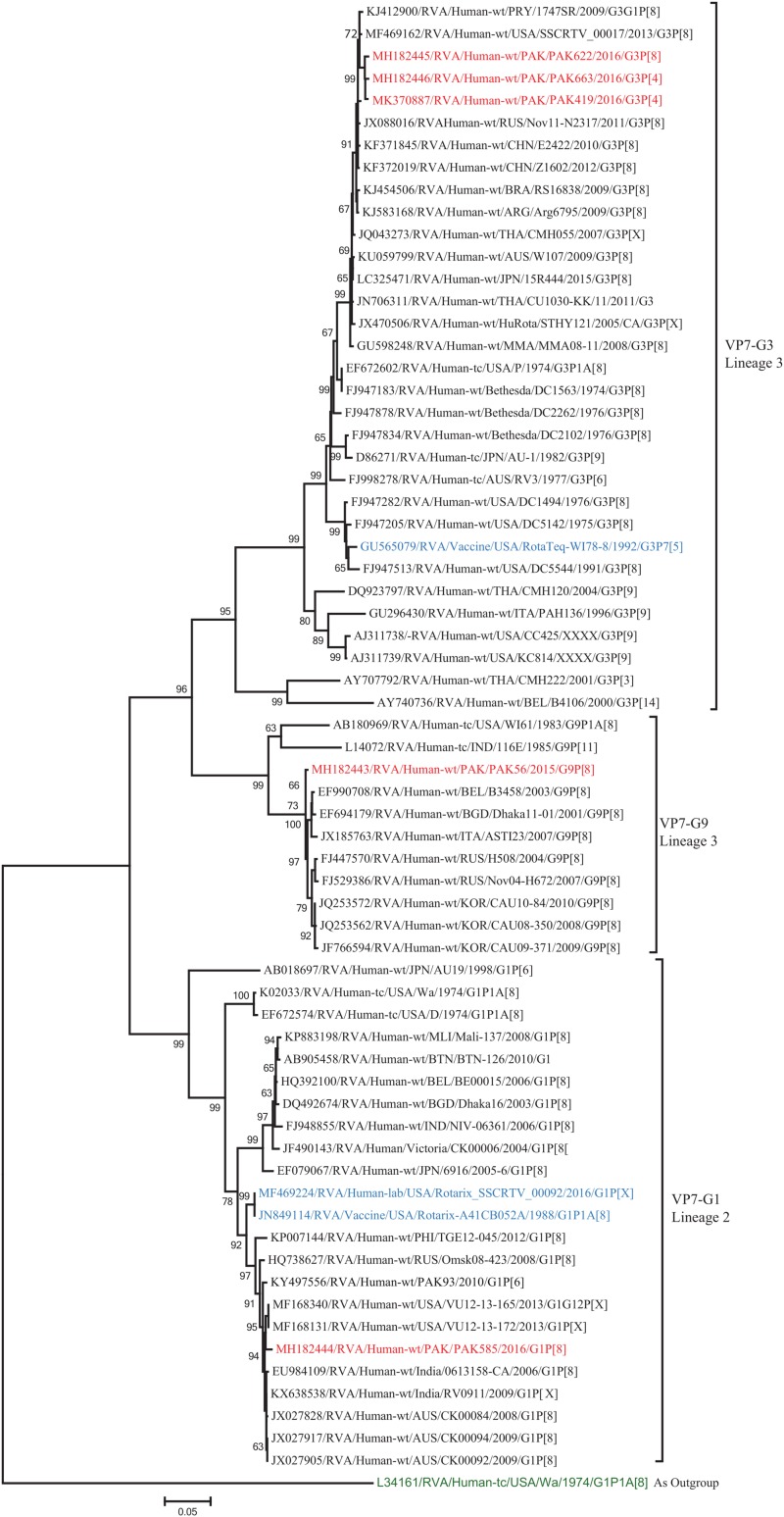
Phylogenetic tree constructed from the nucleotide sequences of the VP7 genes of the study strains and representative RVA strains. RVA strains sequenced in this study are represented by the red color. The vaccine strains and an out group strain are represented by Blue and green color, respectively while black shading represent strains isolated all over the world. The RVA strains sequenced in this study and reference strains obtained from GenBank database are represented by Accession number, Strain Name, Country and year of Isolation. Scale bar: 0.05 substitutions per nucleotide. Bootstrap values <60 are not shown.

The VP7 gene of strain PAK56 clustered closely with G9-lineage three strains from all over the world (Belgium, Bangladesh, Italy, Russia and Korea), and exhibited maximum nucleotide (nt) identities (98.9–99.5%) with G9 strains in Korea (CAU08-350) and Russia (H508). The VP7 gene of strain PAK585 clustered closely with G1-lineage 1 strains from all over the world (India, Australia, United States, Russia, Philippines and previously reported strains from Pakistan), and showed maximum nucleotide (nt) identities (99.3–99.4%) with G1 strain from Australia and India (CK00084, RV0911, respectively). On the other hand, the VP7 genes of strains PAK419, PAK622, and PAK663 clustered together with G3 strains belong to lineage three reported worldwide (United States, Russia, Paraguay, and China), and exhibited highest nucleotide identities (99.2–99.3%) with each other and with G3 strains from Russia (Nov11-N2317) and United States (SSCRTV_00017) (Figure 2).

**FIGURE 3:**
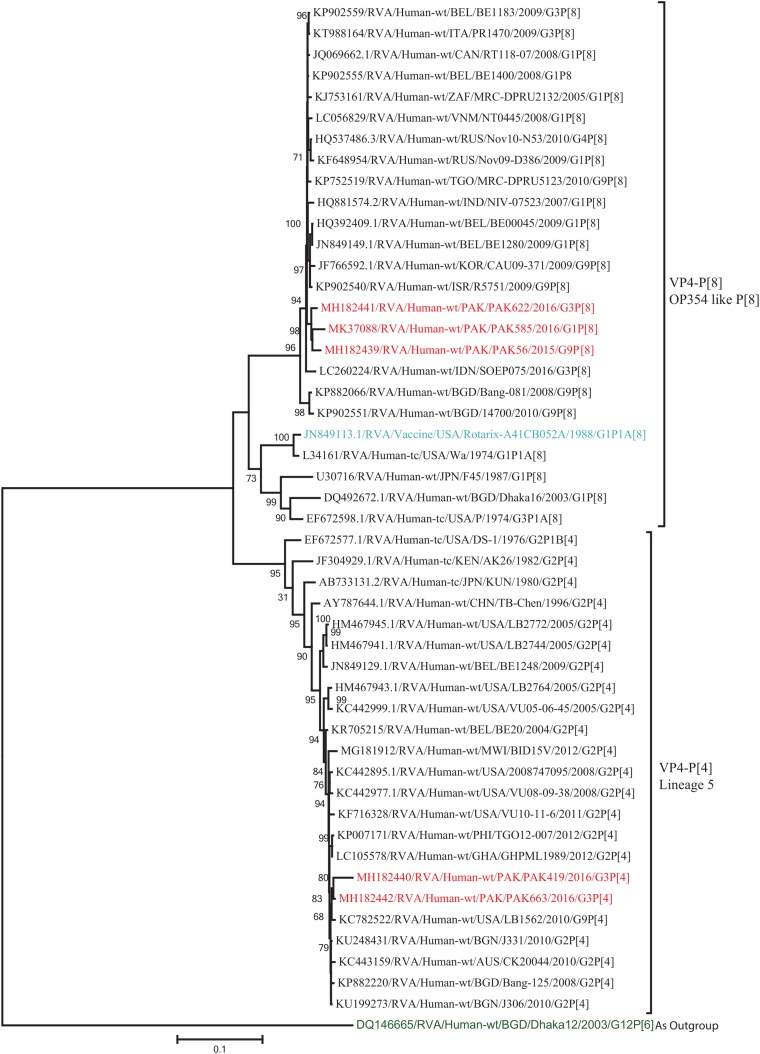
Phylogenetic tree constructed from the nucleotide sequences of the VP4 genes of the study strains and representative RVA strains. RVA strains sequenced in this study are represented by the red color. The vaccine strains and an out group strain are represented by Blue and green color, respectively while black shading represent strains isolated all over the world. The RVA strains sequenced in this study and reference strains obtained from GenBank database are represented by Accession number, Strain Name, Country and year of Isolation. Scale bar: 0.1 substitutions per nucleotide. Bootstrap values <60 are not shown.

The VP4 genes of strains PAK419 and PAK663 clustered closely with other strains identified in P[4] lineage 5 from all over the world (United States, Australia, and Bangladesh), and showed maximum nucleotide identities (98–99.2%) with each other and with DS-1-like strains in United States (LB1562) and Bangladesh (J331) In contrast, VP4 gene of strains PAK56, PAK622, and PAK585 clustered closely with other strains isolated in OP354-like P[8] lineage 4 isolated from other countries of the world, and showed maximum nucleotide identities (98–98.7%) with each other and with P[8] rotavirus strain from Russia (Nov10-N53) and Israel (R5751) (Figure 3).

**FIGURE 4:**
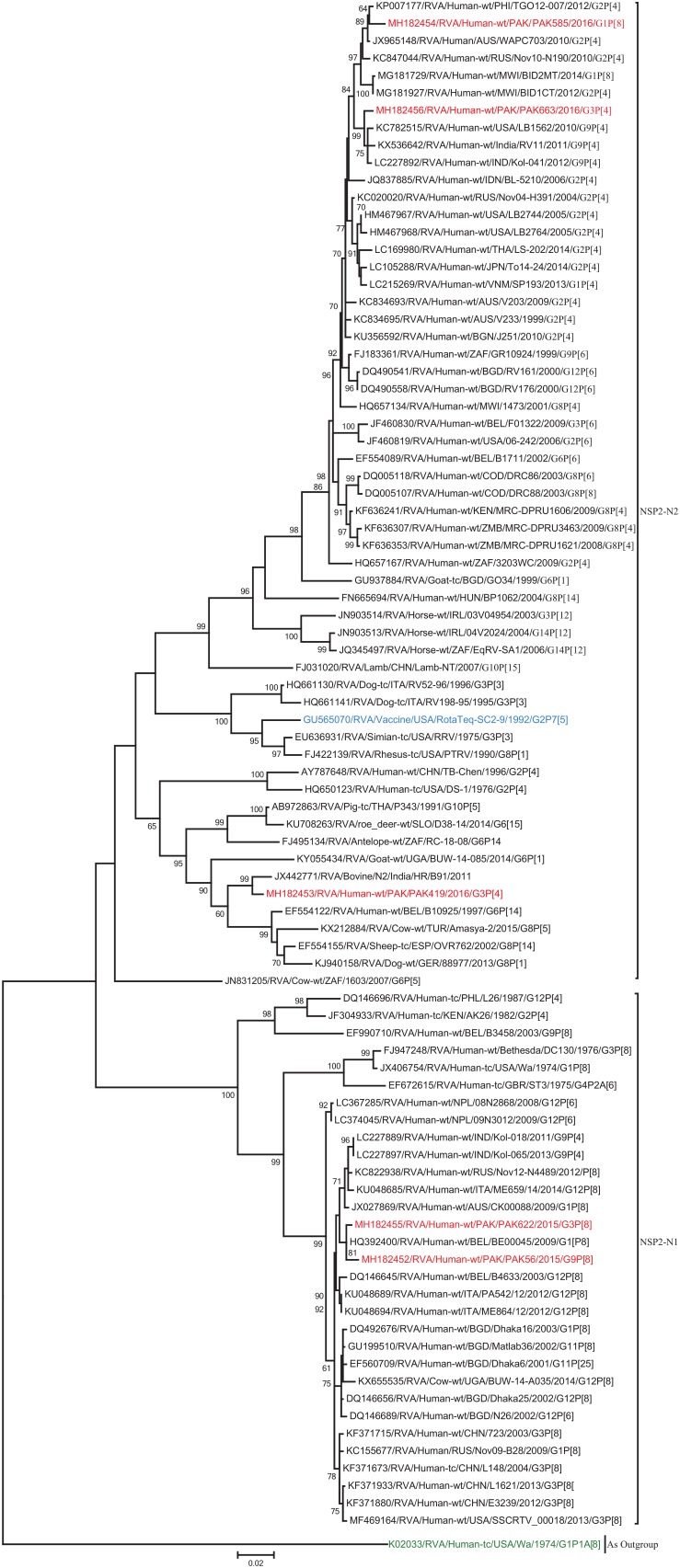
Phylogenetic tree constructed from the nucleotide sequences of the NSP2 genes of the study strains and representative RVA strains. RVA strains sequenced in this study are represented by the red color. The vaccine strains and an out group strain are represented by Blue and green color, respectively while black shading represent strains isolated all over the world. The RVA strains sequenced in this study and reference strains obtained from GenBank database are represented by Accession number, Strain Name, Country and year of Isolation. Scale bar: 0.02 substitutions per nucleotide. Bootstrap values <60 are not shown.

**FIGURE 5:**
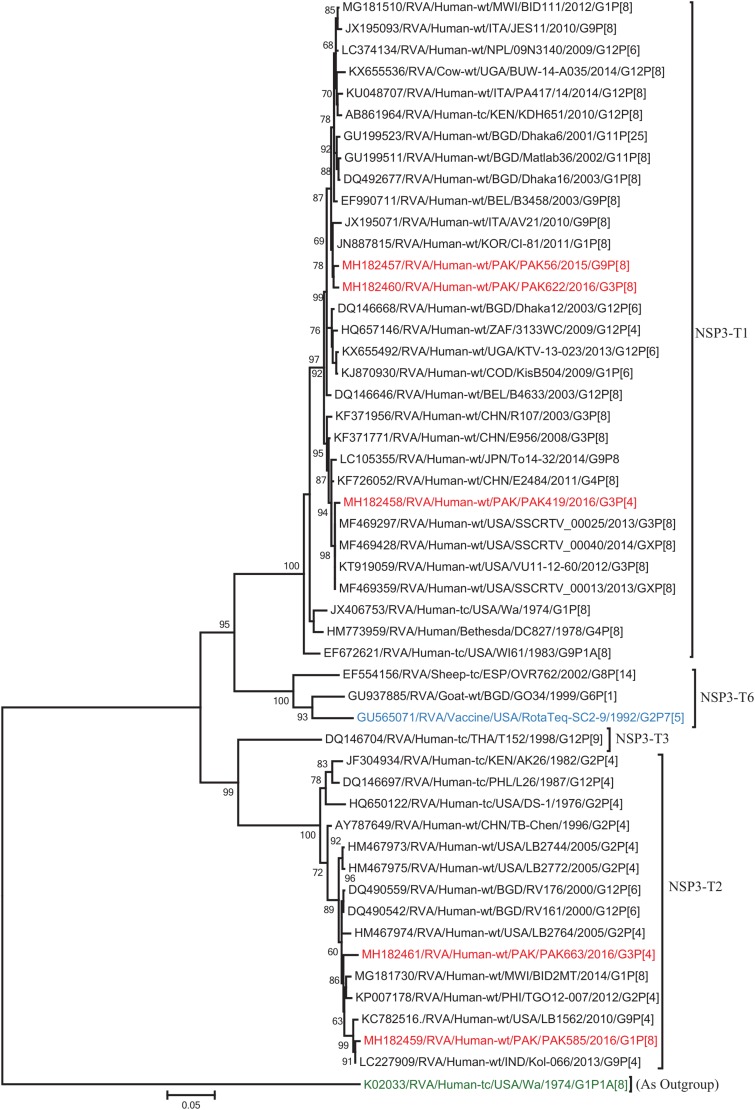
Phylogenetic tree constructed from the nucleotide sequences of the NSP3 genes of the study strains and representative RVA strains. RVA strains sequenced in this study are represented by the red color. The vaccine strains and an out group strain are represented by Blue and green color, respectively while black shading represent strains isolated all over the world. The RVA strains sequenced in this study and reference strains obtained from GenBank database are represented by Accession number, Strain Name, Country and year of Isolation. Scale bar: 0.05 substitutions per nucleotide. Bootstrap values <60 are not shown.

The NSP2 and NSP3 genes of strains PAK56 and PAK622 clustered closely together with other strains reported in NSP2 and NSP3 genotypes N1 and T1, respectively from all over the world (Belgium, Korea, and Italy). The above gene segments of these strains exhibited maximum nucleotide identities (99.4–99.9%) to each other and with Wa-like G1P[8] strains from Belgium (00045) and Korea (C1-81) and comparable identities (97.7–98%) to a cow rotavirus strain from Uganda found in a common branch. The NSP2 gene of strain PAK419 clustered closely together with other globally circulating human and animal rotavirus strains identified in NSP2 genotype N2, and showed maximum nucleotide (nt) identity (98.3%) with Indian bovine strain (HR/B91). While, NSP3 gene segment of PAK419 clustered closely together with strains reported worldwide in NSP3 genotype T1, and showed 98.9% nucleotide identity with Chinese Wa-like strain (E2484/G4P[8]). In contrast, the NSP2 and NSP3 genes of strains PAK585 and PAK663 clustered closely together with other strains identified in previously established genotypes of NSP2 and NSP3 (N2 and T2, respectively) from all over the world (Philippines, United States, India, Bangladesh, and Malawi), and showed nucleotide identities of (96.4–97.3%) and (98–99.5%) with each other and with that of DS-1 like strains from the Philippines (TGO12-007), United States (LB1562), India (KOL-006), respectively (Figures 4, 5).

**FIGURE 6:**
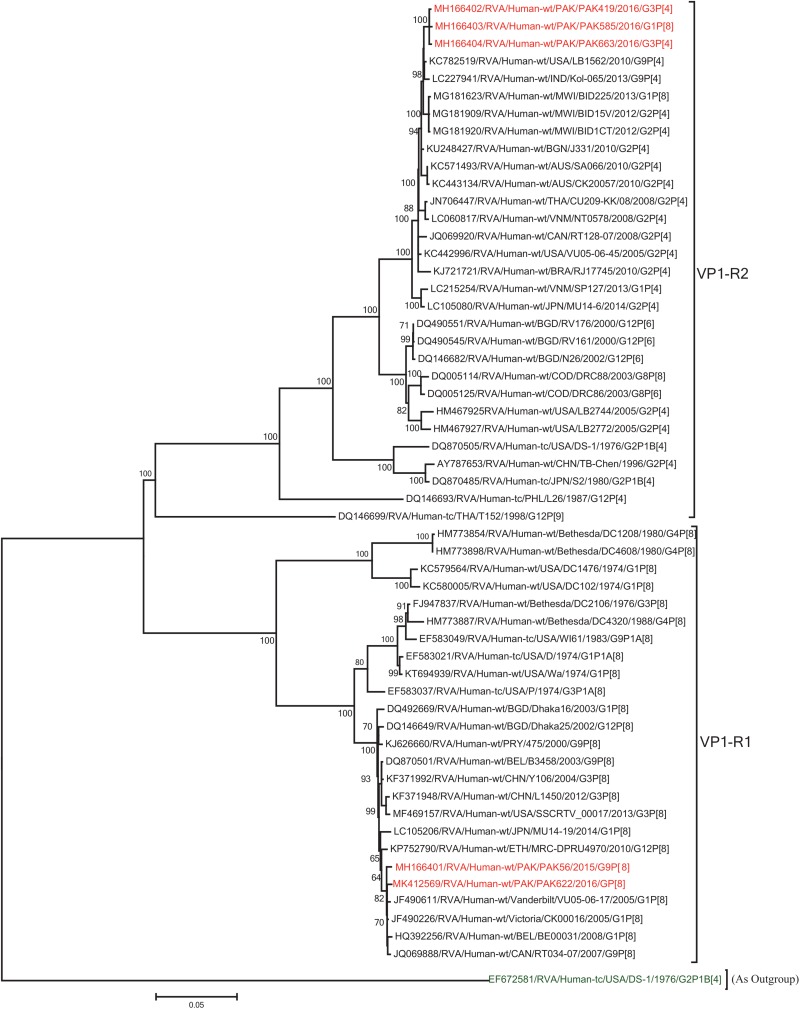
Phylogenetic tree constructed from the nucleotide sequences of the VP1 genes of the study strains and representative RVA strains. RVA strains sequenced in this study are represented by the red color. The vaccine strains and an out group strain are represented by Blue and green color, respectively while black shading represent strains isolated all over the world. The RVA strains sequenced in this study and reference strains obtained from GenBank database are represented by Accession number, Strain Name, Country and year of Isolation. Scale bar: 0.05 substitutions per nucleotide. Bootstrap values <60 are not shown.

**FIGURE 7:**
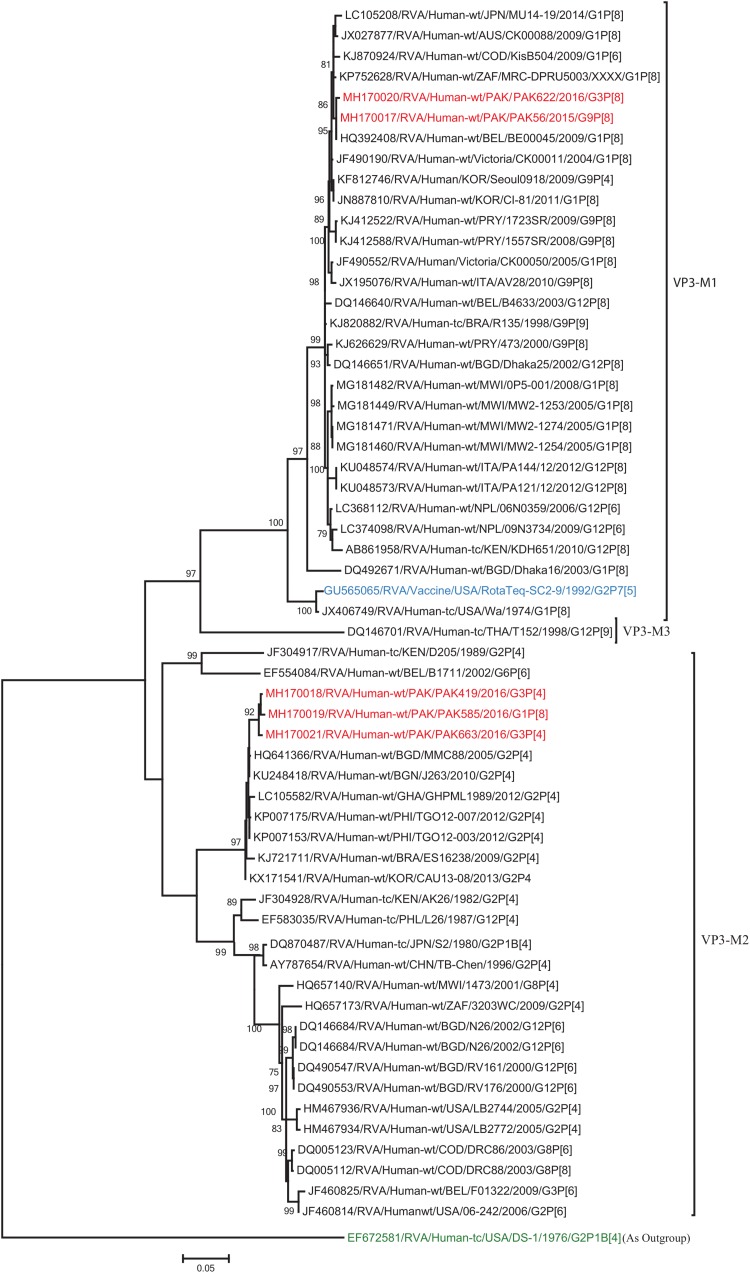
Phylogenetic tree constructed from the nucleotide sequences of the VP3 genes of the study strains and representative RVA strains. RVA strains sequenced in this study are represented by the red color. The vaccine strains and an out group strain are represented by Blue and green color, respectively while black shading represent strains isolated all over the world. The RVA strains sequenced in this study and reference strains obtained from GenBank database are represented by Accession number, Strain Name, Country and year of Isolation. Scale bar: 0.05 substitutions per nucleotide. Bootstrap values <60 are not shown.

**FIGURE 8:**
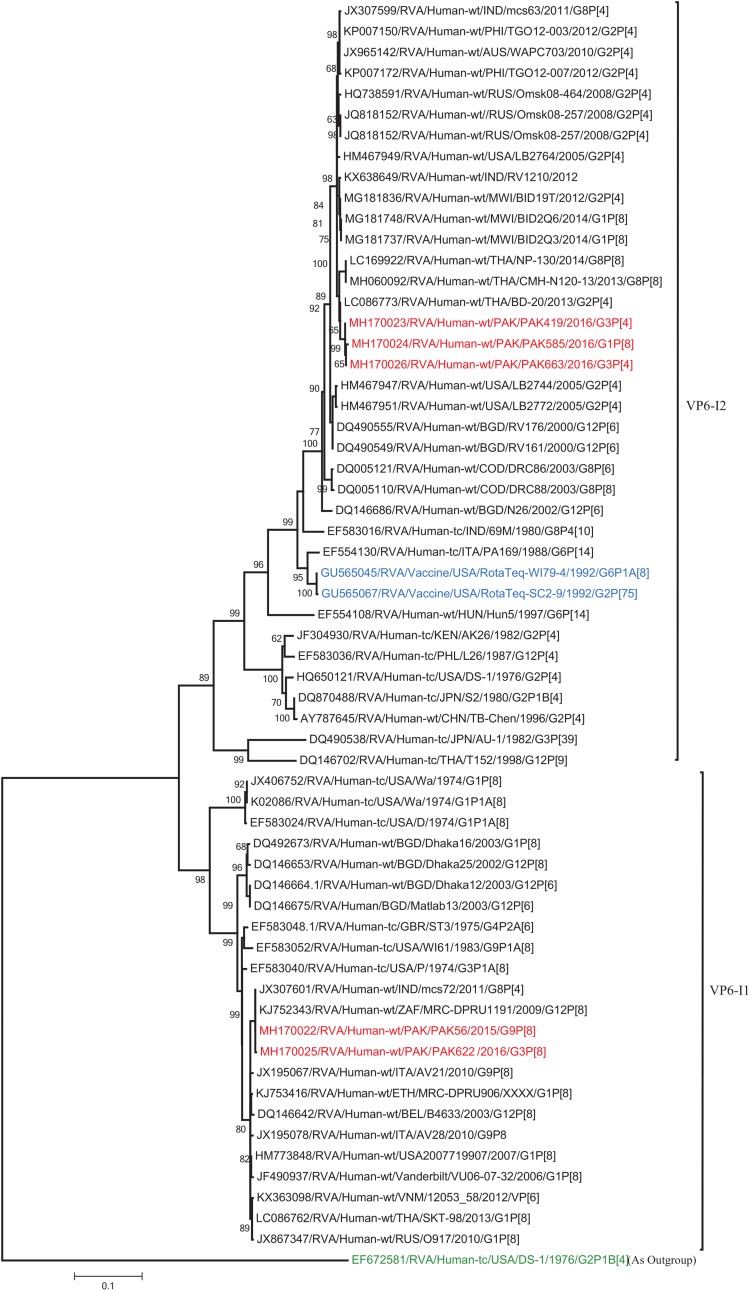
Phylogenetic tree constructed from the nucleotide sequences of the VP6 genes of the study strains and representative RVA strains. RVA strains sequenced in this study are represented by the red color. The vaccine strains and an out group strain are represented by Blue and green color, respectively while black shading represent strains isolated all over the world. The RVA strains sequenced in this study and reference strains obtained from GenBank database are represented by Accession number, Strain Name, Country and year of Isolation. Scale bar: 0.1 substitutions per nucleotide. Bootstrap values <60 are not shown.

**FIGURE 9:**
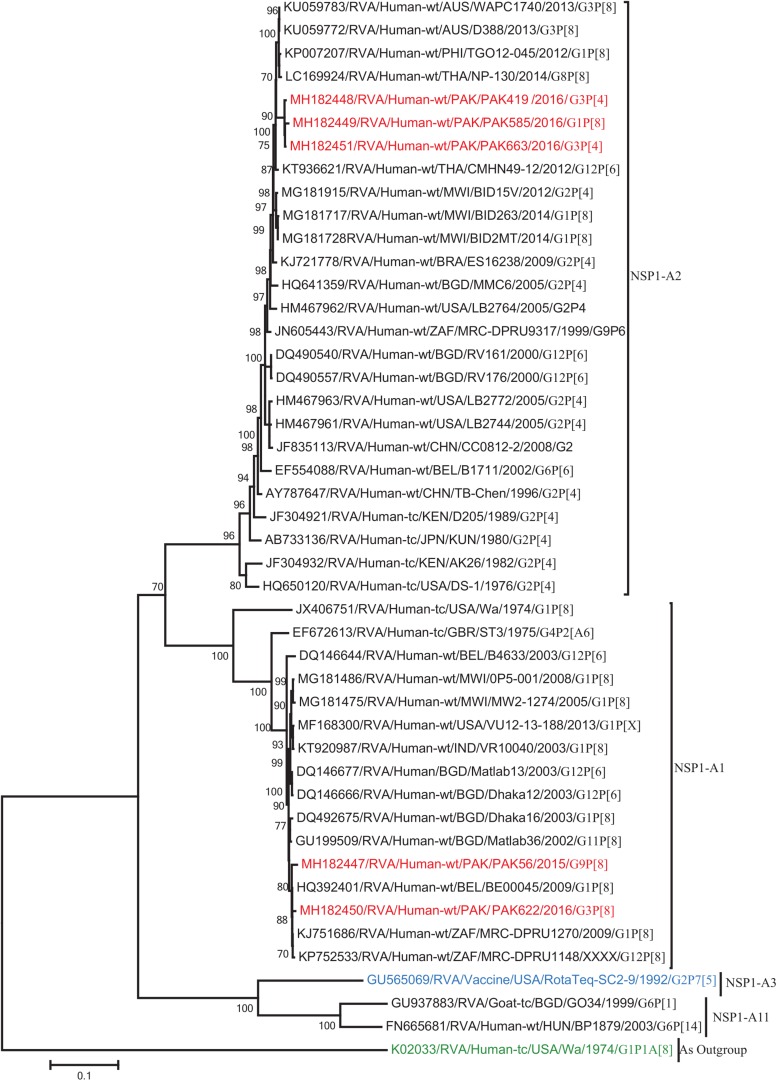
Phylogenetic tree constructed from the nucleotide sequences of the NSP1 genes of the study strains and representative RVA strains. RVA strains sequenced in this study are represented by the red color. The vaccine strains and an out group strain are represented by Blue and green color, respectively while black shading represent strains isolated all over the world. The RVA strains sequenced in this study and reference strains obtained from GenBank database are represented by Accession number, Strain Name, Country and year of Isolation. Scale bar: 0.1 substitutions per nucleotide. Bootstrap values <60 are not shown.

**FIGURE 10:**
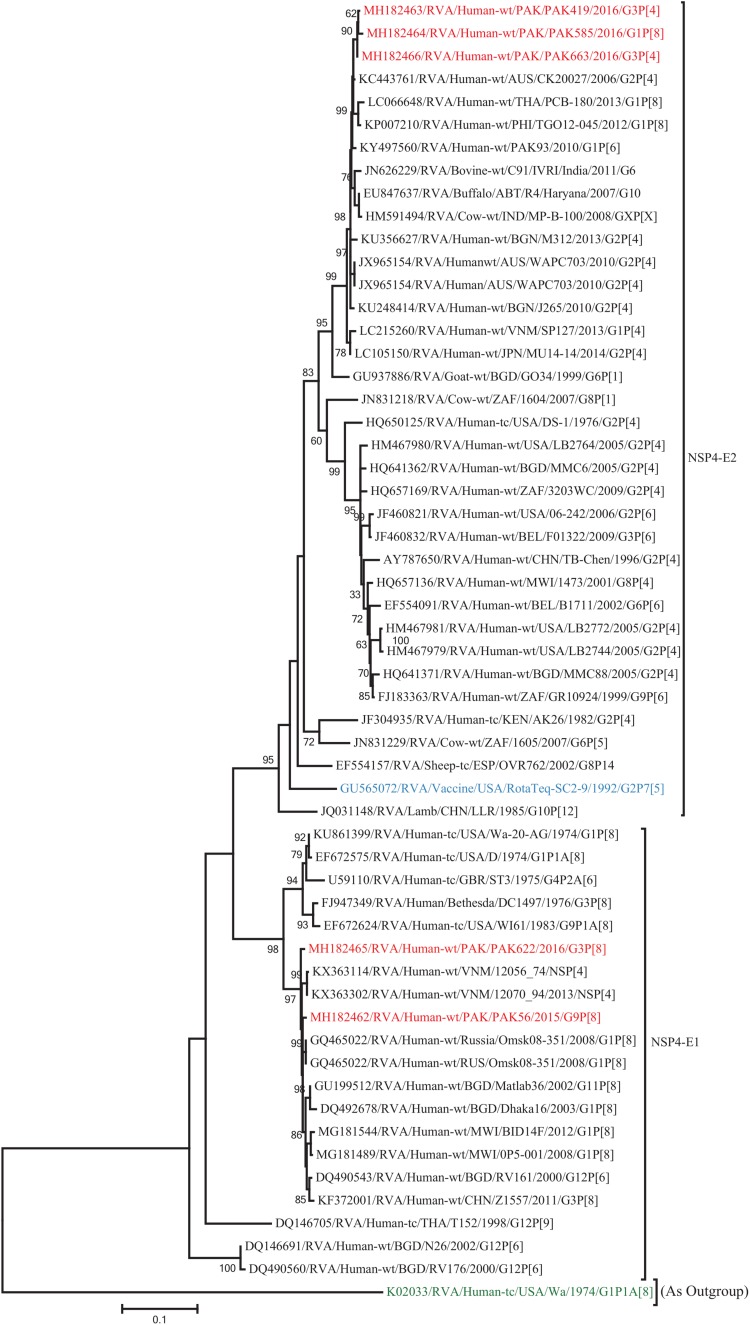
Phylogenetic tree constructed from the nucleotide sequences of the NSP4 genes of the study strains and representative RVA strains. RVA strains sequenced in this study are represented by the red color. The vaccine strains and an out group strain are represented by Blue and green color, respectively while black shading represent strains isolated all over the world. The RVA strains sequenced in this study and reference strains obtained from GenBank database are represented by Accession number, Strain Name, Country and year of Isolation. Scale bar: 0.5 substitutions per nucleotide. Bootstrap values <60 are not shown.

**FIGURE 11:**
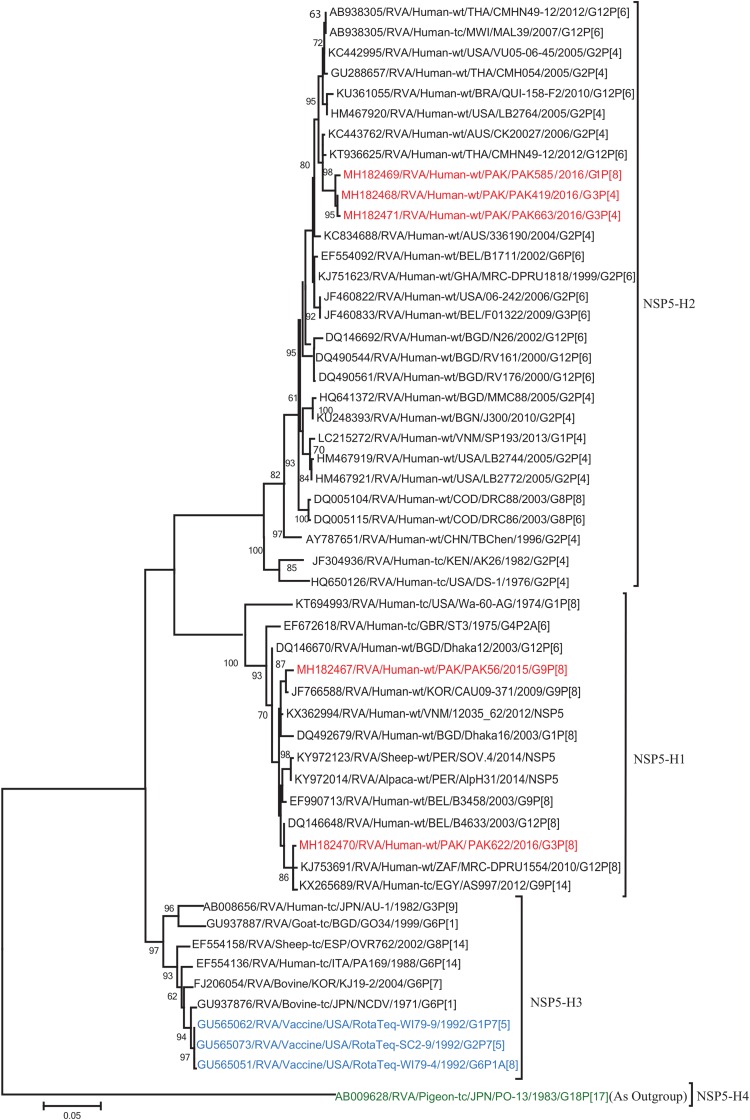
Phylogenetic tree constructed from the nucleotide sequences of the NSP5 genes of the study strains and representative RVA strains. RVA strains sequenced in this study are represented by the red color. The vaccine strains and an out group strain are represented by Blue and green color, respectively while black shading represent strains isolated all over the world. The RVA strains sequenced in this study and reference strains obtained from GenBank database are represented by Accession number, Strain Name, Country and year of Isolation. Scale bar: 0.05 substitutions per nucleotide. Bootstrap values <60 are not shown.

The VP1, VP3, VP6, NSP1, NSP4, and NSP5 gene segments of three of our strains (PAK419, PAK585, and PAK663) phylogenetically clustered closely together with strains circulating globally (United States, Belgium, Thailand, Bangladesh, and Australia) in the previously established genotypes R2, M2, I2, A2, E2, H2, respectively. These six genotypes of three strains showed maximum nucleotide similarities (99.3–99.9%) to each other and (98.2.8–99.5%) to the DS-1-like rotavirus strains detected in United States (LB1562), Bangladesh (MMC-88) Australia (CK20027), Thailand (BD-20, CMHN49-12 and CMHN49-12). In contrast the VP1, VP3, VP6, NSP1, and NSP4 gene segments of two of our strains (PAK56 and PAK622) clustered closely together with other globally reported strains (United States, Belgium, India, Russia, and South Africa) in the genotypes R1, M1, I1, and E1, respectively. These five genotypes of strains PAK56 and PAK622 showed maximum nucleotide similarities (98.9–100%) to each other and to the Wa-like strains from United States (Vanderbilt/VU-05-06-17), Belgium (BE00045), India (mcs72), South Africa (ZAF/MRC-DPRU1191) and Russia (Omsk08-351). While, gene segment NSP5 of PAK56 and PAK622 clustered with other rotavirus strains detected round the globe (Korea, Bangladesh, Africa, and Peru) in the genotype H1 and showed maximum nucleotide (nt) similarities of (99.4–99.8%) to rotavirus strains detected in Korea (CAU/09-371) and Africa (ZAF/MRC-DPRU1554) and comparable identities (97.9 and 98.3%, respectively) with sheep (PER/SOV.4) and Alpaca (PER/AlpH31) rotavirus strains from Peru in the same cluster (Figures 6–11).

**FIGURE 12:**
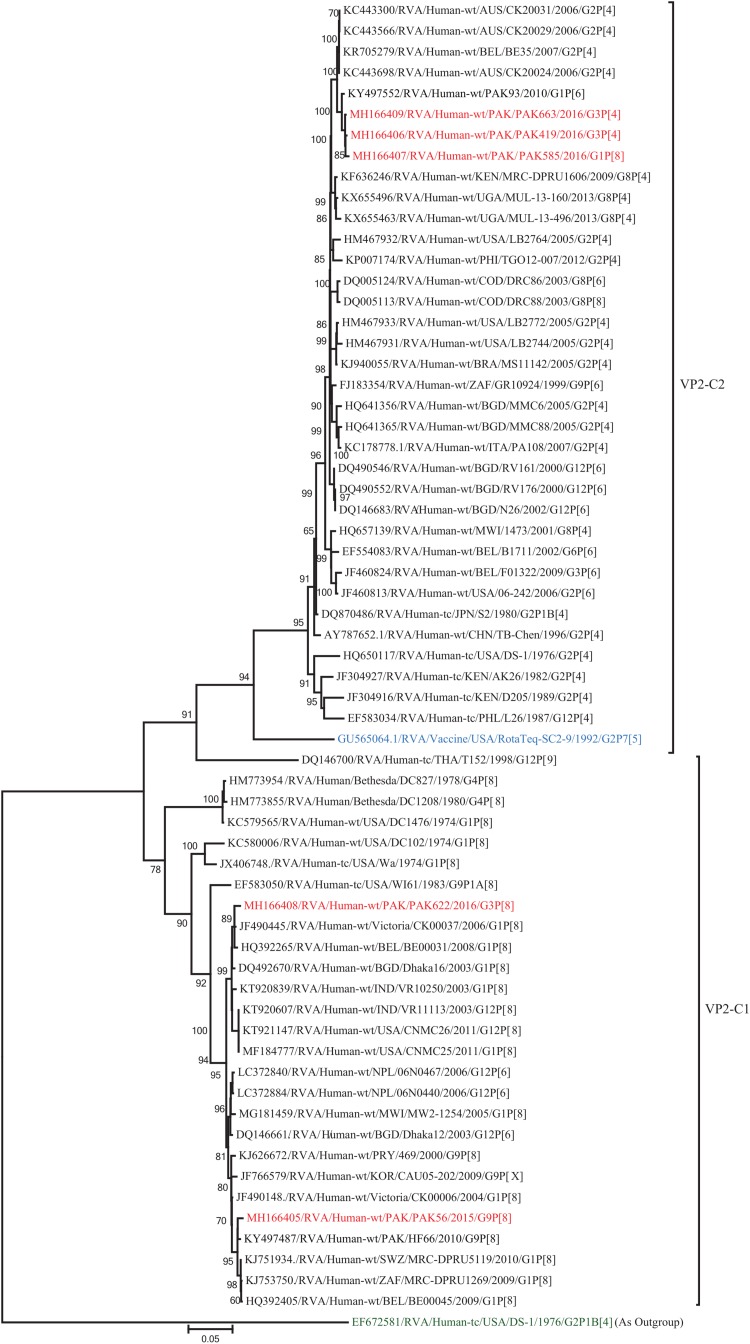
Phylogenetic tree constructed from the nucleotide sequences of the VP2 genes of the study strains and representative RVA strains. RVA strains sequenced in this study are represented by the red color. The vaccine strains and an out group strain are represented by Blue and green color, respectively while black shading represent strains isolated all over the world. The RVA strains sequenced in this study and reference strains obtained from GenBank database are represented by Accession number, Strain Name, Country and year of Isolation. Scale bar: 0.05 substitutions per nucleotide. Bootstrap values <60 are not shown.

The VP2 genes of strains PAK419, PAK585, and PAK663 clustered closely together with other strains identified in VP2 lineage C2 from all over the world (Belgium, Australia and previously reported strain from Pakistan), and exhibited maximum nucleotide similarities (99.1–99.2%) with co-circulating DS-1-like strain from Pakistan (PAK93) and comparable identities (98.5–98.7%) with Australian DS-1-like strain (CK20024/G2P[4]). On the other hand, the VP2 gene of strains PAK56 and PAK622 clustered with other strains identified in VP2 lineage C1 from all over the world (Belgium, Bangladesh, India, Africa, United States, Australia, Korea, Italy, Egypt, Vietnam, and Pakistan) and showed maximum nucleotide identities (99.1–99.3%) with Wa-like strains from Australia (Victoria/CK00037) and Belgium (BE00031) and previously reported strain from Pakistan (HF66) (Figure 12).

## Discussion

In the present study, the whole genomes of five human RVA strains (PAK56, PAK419, PAK585, PAK622, and PAK663) were characterized for the first time in children with severe gastroenteritis in Pakistan in 2015–2016. The two strains (PAK56 and PAK622) exhibited a typical Wa-like backbone while, three strains (PAK419, PAK585, and PAK663) showed genotype constellation involving genogroup 1 and genogroup 2 genes. The unique genotype constellation of (PAK419, PAK585, and PAK663) were found to be similar to DS-1-like intergenogroup reassortant strains. Pakistan has implemented RVA vaccine in national immunization program in January 2018, so the emergence of intergenogroup reassorted strains in the present study are not linked to RVA vaccine introduction.

The DS-1-like G1P[8] double-gene reassortant strains were first detected in Japan followed by cases reported in Thailand, Vietnam, Malawi, and Brazil (Komoto et al., 2010, 2016; Nakagomi et al., 2012; Fujii et al., 2014; Kuzuya et al., 2014; Yamamoto et al., 2014; Jere et al., 2018; Luchs et al., 2019). The unusual DS-1-like G1P[8] double-gene reassortant strain PAK585 detected in the present study showed close similarity with other DS-1-like, DS-1-like G1P[8] and Wa-like strains circulating globally (India, Australia, United States, Thailand, Bangladesh, Malawi, and Philippines), as observed in the phylogenetic tree. The analysis of PAK585 revealed that eight out of eleven gene segments (VP1-VP3, VP6, NSP1-NSP3, and NSP5) of this strain showed close similarities with DS-1-like intergenogroup reassorted strains or typical DS-1 like strains circulating worldwide. One segment (NSP4) showed close nucleotide similarities (97.6–98.2%) with human strains from Australia, Thailand, Philippines, and previously reported strains from Pakistan. While the remaining two segments (VP7 and VP4) were assumed to be derived from typical human Wa-like strains circulating worldwide. Therefore, the emergence of this unusual Pakistani G1P[8] strain more likely was due to their spread from other Asian countries into Pakistan, rather than the local emergence through multiple reassortment event(s) between locally circulating Ds-1-like and Wa-like G1P[8] strains.

The G3 genotype is documented as the third most predominant RVA genotype in humans mostly found in combination with P[8] (Atima et al., 2016). The G3 rotavirus strains has wide host range comprising cows, dogs, cats, horses rabbits, pigs, bats, sheeps, and monkeys in association with many P genotypes (Matthijnssens et al., 2011a). In this study we have detected two strains possessing the G3P[4] genotype, a single gene reassortant strain (PAK663/G3P[4]) and double gene reassortant starin (PAK419/G3P[4]). These two strains showed close relationship with G2P[4], G3P[8], G9P[4], and G12P[6] strains identified worldwide (United States, Thailand, India, Bangladesh, Malawi, Philippines, and Australia) and previously reported G1P[6] strain in Pakistan. These unusual G3 strains shared nine gene segments (VP1-VP4, VP6, NSP1-NSP2, and NSP4-NSP5) with typical DS-1-like or DS-1-like intergenogroup reassortant strains, whereas, one segment (VP7) was assumed to be of typical human Wa-like in the phylogenetic tree. Notably, NSP3 gene of strain PAK419 was observed with an additional reassortment event in which T2 genotype of strain PAK419 was replaced with human Wa-like genotype T1. So, it is suggested that strain PAK419 and PAK663 have been derived in Pakistan due to multiple reassortments events between locally and globally circulating human Ds-1-like, Wa-Like, human like bovine or bovine strains. Moreover, on phylogenetic observation, PAK663 and PAK 419 strains were found to be close to each other in their maximum gene segment, suggesting they have evolved from a common ancestor.

On the other hand, two Pakistani strains PAK56 and PAK622 having genotypes G9P[8] and G3P[8] detected in the present study were found to be very similar to a typical human Wa-like strains reported in several countries worldwide. The analysis of ten of the eleven gene segments (VP1-VP4, VP6-VP7, NSP1-NSP4) of strains PAK56 and PAK 622 showed a typical human Wa-like origin. While, gene segment NSP5 showed close relationship with human rotavirus strains detected in Korea and Africa and with sheep and Alpaca rotavirus strains from Peru in the same cluster. These observation suggests that interspecies transmission and reassortments might have occurred between human/caprine rotavirus strains. Also on the phylogenetic tree, these two strains were clustering closely in their maximum gene segments suggestive of their origin from a common ancestor.

## Conclusion

This is the first full genome characterization of human rotavirus strains circulating in Pakistan. The evolution of DS-1-like intergenogroup reassortant strains having G1P[8] and G3P[4] genotypes in Pakistan suggested the constant circulation of these unusual rotavirus strains and ongoing reassortment associated with them particularly in Asia. After WHO recommendation in 2009, the universal implementation of RVA vaccine is continue to increase globally. Whether or not the coinciding rise of strains with Wa-like VP4 and VP7 genes together with a DS-1-like backbone and mass vaccine introduction is consequential or purely random remains enigmatic. Therefore, further investigations via continuous surveillance of these strains is recommended to monitor the evolution, spread and genetic stability of novel reassortant rotavirus strains derived from such events. Pakistan has licensed RVA vaccine in national EPI program in January 2018. After the introduction of RVA vaccine in the country’s immunization program the careful monitoring is needed to estimate vaccine effectiveness and challenges to current vaccine strategies against such unusual strains.

## Data Availability Statement

The datasets generated for this study can be found in the nucleotide sequences were submitted to the GenBank under the following accession numbers: (VP7) MH182443–MH182446 and MK370887, (VP4) MH182439–MH182442 and MK370888, (VP6) MH170022–MH170026, (VP1) MH166401–MH166404 and MK412569, (VP2) MH166405–MH166409, (VP3) MH170017–MH170021, (NSP1) MH182447–MH182451, (NSP2) MH182452–MH182456, (NSP3) MH182457–MH182461, (NSP4) MH182462–MH182466, (NSP5) MH182467–MH182471.

## Ethics Statement

The studies involving human participants were reviewed and approved by the Ethical Committee of PIMS, Benazir Bhutto Shaheed Hospital (BBH) and Internal Review Board (IRB) of COMSATS Institute of Information Technology. Written informed consent to participate in this study was provided by the participants’ legal guardian/next of kin. Written informed consent was obtained from the minor(s)’ legal guardian/next of kin for the publication of any potentially identifiable images or data included in this article.

## Author Contributions

AS and NB conceived concept and designed the experiments. AS performed the experiments and wrote the manuscript. NB, HB, KY, and JM contributed the reagents and data analysis tools and helped in write-ups.

## Conflict of Interest

The authors declare that the research was conducted in the absence of any commercial or financial relationships that could be construed as a potential conflict of interest.

## Footnotes

1 http://www.ncbi.nlm.nih.gov/orffinder
